# Turning Unpredictable Biomolecule Adsorption to Controlled Corona Formation: Focus on Carbon Nanomaterials

**DOI:** 10.1002/adma.202523328

**Published:** 2026-04-18

**Authors:** Yajuan Zou, Yalei Hu, Jie Yu, Naoki Komatsu, Yuta Nishina, Alberto Bianco

**Affiliations:** ^1^ Research Institute for Interdisciplinary Science Okayama University Okayama Japan; ^2^ CNRS Immunology Immunopathology and Therapeutic Chemistry University of Strasbourg ISIS Strasbourg France; ^3^ Graduate School of Human and Environmental Studies Kyoto University Kyoto Japan

**Keywords:** carbon dots, carbon nanotubes, graphene, nanodiamonds, proteins

## Abstract

With unique optical and physicochemical properties, carbon nanomaterials (CNMs), including carbon nanotubes, graphene‐related materials, nanodiamonds, and carbon dots, are extensively explored as platforms for cancer diagnosis and treatment. However, in biofluids, CNMs spontaneously adsorb biomolecules to form an unpredictable corona, obstructing the implementation of their designed functions. In this review, we summarize how the intrinsic and acquired properties of CNMs affect protein corona formation, and the consequent biological and toxicological outcomes, as well as strategies to reshape the composition and structural organization of adsorbed proteins. This comprehensive knowledge will provide insights into developing CNMs with tailored corona and requested functions in cancer nanomedicine, advancing their translations into clinics.

## Graphical abstract

Controlling the protein corona formation onto carbon nanomaterials (CNMs) enhances their functionalities as platforms for cancer theranostics. Here, we reviewed the effects of the intrinsic and acquired properties of CNMs on protein corona formation, the consequent biological and toxicological outcomes, and the strategies to reshape corona formation.

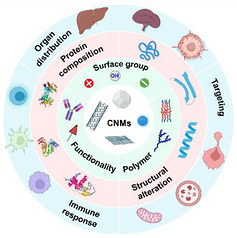

## 1 Introduction

As most biological events occur at the nanoscale level, nanotechnology offers a myriad of opportunities in nanomedicine, especially for cancer diagnosis and treatment [1]. Whilst being small enough to facilitate blood vessel transportation, engineered nanomaterials (ENMs) with tunable shapes and surface chemistries can induce specific interactions with targeted cells for precise therapy [2]. However, owing to their large surface area and high surface energy, ENMs spontaneously adsorb proteins in the biological milieu to form a so‐called “protein corona” [3]. Of note, the competitive binding between ENMs and proteins creates a transient protein corona that endures dynamic evolutions [4, 5]. Initially, highly abundant proteins (e.g., albumin and immunoglobulin) quickly adhere to ENMs and dominate the particle surface for a short period. Afterward, the weakest bound proteins are gradually dissociated from ENMs and displaced by those with less abundance but higher affinity (e.g., apolipoprotein and complement proteins). This competitive protein adsorption phenomenon, known as the Vroman effect [6, 7], occurs not only over time [8], but also during the translocation of ENMs from one physiological compartment to another [9]. Among the adsorbed proteins, those bound tightly to ENMs constitute an inner layer of hard protein corona [10]. In contrast, the loosely attached proteins that undergo rapid exchanges are forming the outer layer of soft corona. Due to the fast dissociation rate of soft corona proteins [11], it is technically challenging to capture and isolate these proteins for analysis [12]. Thereby, the current understanding of the biological identity of protein corona is mostly limited to the hard corona proteins.

The adsorbed proteins not only shield the synthetic identity of ENMs, but also confer a new biological identity to these materials, affecting the interactions of ENMs with biological systems [13, 14]. Accordingly, cellular uptake and subcellular localization, in vivo fates including pharmacokinetics, biodistribution, therapeutic efficacy, and biocompatibility of ENMs are largely dependent on the composition of protein corona as well as the structural organization and conformational arrangement of bound proteins [15]. The formation of protein corona is a complicated process driven by hydrophobic and electrostatic interactions, hydrogen bonding, and van der Waals forces at the interface between ENMs and proteins [16]. As a result, both the properties of ENMs, including size, shape, and surface chemistry [17], and the molecular traits of proteins, such as polypeptide sequence, secondary structure, and three‐dimensional conformation, affect their association with ENMs [10], leading to unpredictable and uncontrolled protein corona formation.

As the protein corona is the biological identity of ENMs that dictates their biological performance, controlling the formation of such a corona allows ENMs to function better. At the early stage of protein corona research, immunoglobulin and complement proteins as main components in the corona layer were reported to initiate the phagocytic process of ENMs by macrophages in the reticuloendothelial system (RES), resulting in their rapid clearance from systemic circulation and in a decreased delivery efficacy to target sites [18, 19]. Consequently, substantial efforts were devoted to prevent protein corona formation by functionalizing ENMs with stealth surface coatings such as poly(ethylene glycol) (PEG) and zwitterionic polymers [20]. However, this trend was challenged by the breakthrough finding that the adsorption of apolipoprotein J, a lipoprotein belonging to dysopsonin protein family, was required for conferring stealth effect to ENMs [21]. Since then, research interests have been shifted from avoiding protein corona formation to turning the composition of protein corona. A similar trend was observed in the research interest toward the targeting ability of ENMs. Since the disclosure that transferrin loses its targeting specificity in the presence of a protein corona [22], there has been an unchangeable belief that protein corona would compromise or even nullify the targeting moieties immobilized on ENM surface. However, it has been demonstrated that the antibodies preserved their targeting ability in protein‐rich biofluids when they were combined with ENMs via a non‐covalent adsorption, rather than through a covalent bonding [23]. Indeed, the adsorption approach enables the effective orientation of the antigen‐binding domain toward its corresponding antigen, while chemical modification disturbs the structure of the antibody to result in an unfavorable conformational rearrangement. These findings not only brought a paradigm shift to the design of nano‐formulations with required targeting ability but also highlight the importance of controlling the orientation of functional epitopes or cellular recognition motifs of adsorbed proteins.

Nonetheless, these achievements are mainly accomplished through investigations on soft organic nanomaterials, including lipid‐based and polymeric nanoparticles, which are the main bodies of clinically approved or relevant nano‐formulations [24]. Carbon‐based nanomaterials (CNMs), on the other hand, also demonstrated high potential for cancer theranostics owing to their high biocompatibility, unique optical and photothermal properties, and easiness of controlled surface modification [25]. However, the clinical application of CNMs is still under development. Although several reviews have correlated the protein corona formed on a specific type of CNM to the targeting ability, and/or distribution in animal models [26, 27, 28, 29], such scattered knowledge is yet to be explored toward the precise prediction of the behavior of CNMs in human body. Systematic reviewing of unpredictable protein corona on CNMs to achieve controlled corona formation holds great promise in bridging the translational gap between laboratory studies of CNMs and practical applications for patient therapy and diagnosis.

**FIGURE 1:**
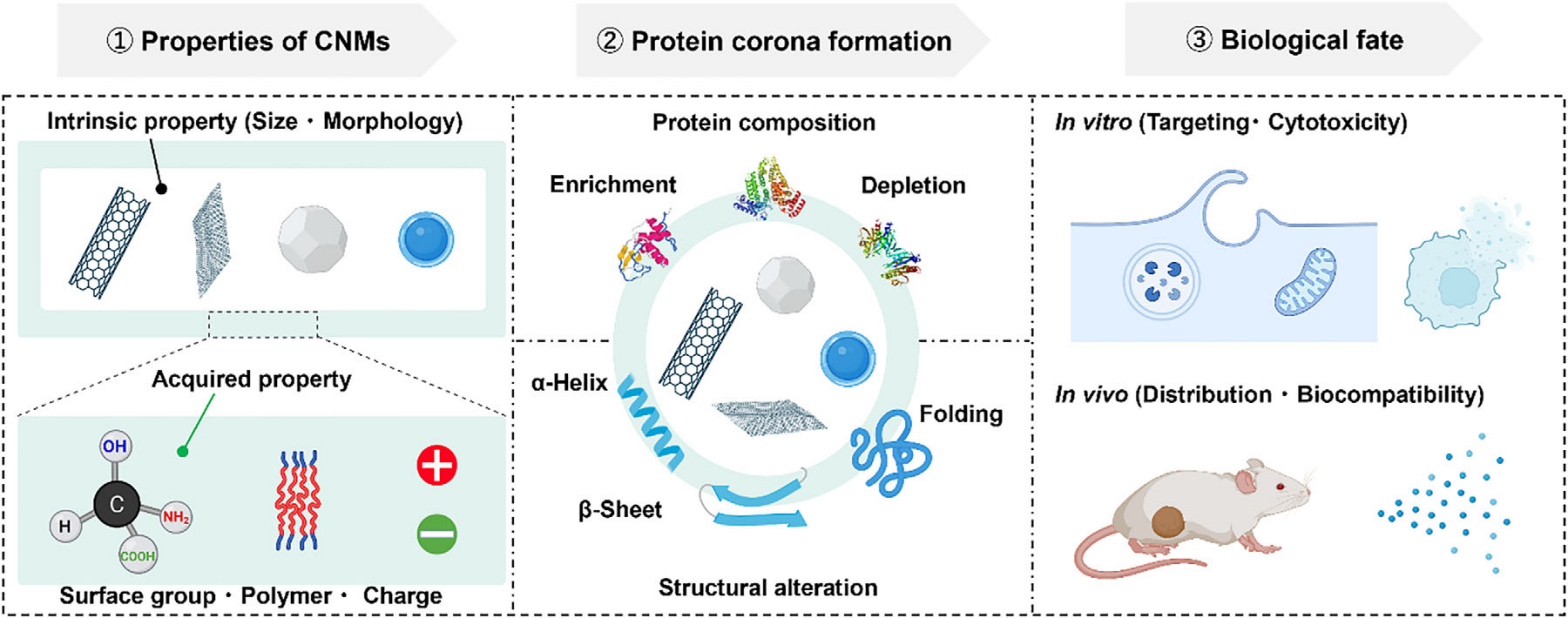
Understanding the interactions between CNMs and proteins, rethinking the effects of CNM properties on protein corona formation and their biological fate, and in turn guiding the rational design of CNMs for customizing corona formation toward satisfactory efficacy and safety.

In this review, focusing on four most intensively investigated CNMs, which include carbon nanotubes (CNTs), graphene‐related materials (GRMs), nanodiamonds (NDs), and carbon dots (CDs), we will discuss how their intrinsic and acquired properties affect protein corona formation, and the consequent biological and toxicological outcomes (Figure 1). The evolution of protein corona from one biological compartment to another will be discussed as protein corona formation is a dynamic process. In addition to tailoring the composition of protein corona at macroscopic level, we will present microscopic‐level information about tuning the conformation of proteins adsorbed onto CNTs and graphene. At the outlook, we will give perspectives on information‐assisted CNM synthesis and protein engineering for tailored protein corona formation, in an attempt to accelerate the development of CNMs for precise cancer theranostics, as well as to advance their translation to clinics.

## 2 Carbon Nanotubes

**FIGURE 2:**
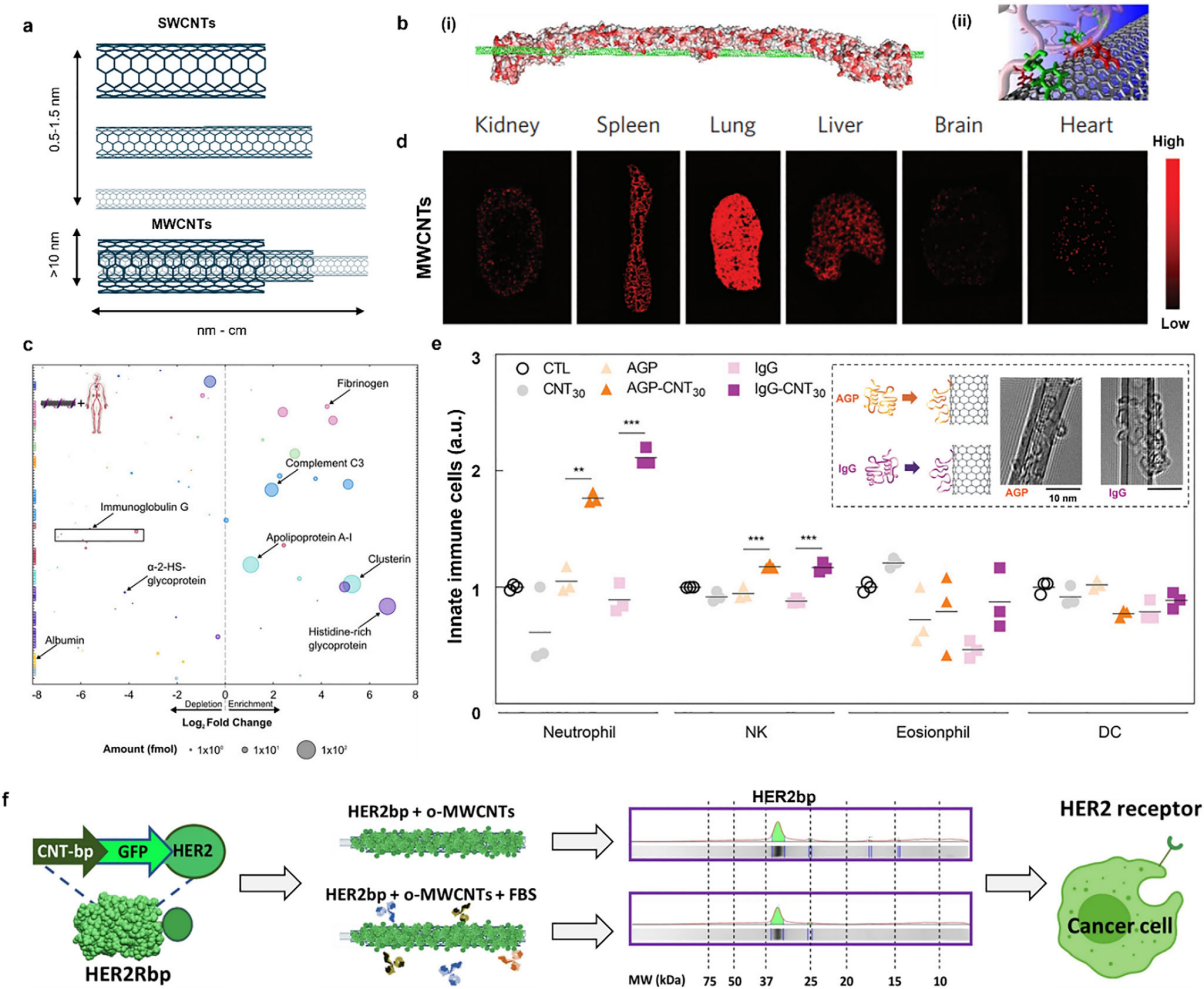
(a) Schematic representation of SWCNTs and MWCNTs. (b) The interactions of fibrinogen (i) [36] and the aromatic residues from fibrinogen (ii) [37] with SWCNTs. Reproduced with permission. Copyright 2016, Springer Nature; Copyright 2011, National Academy of Sciences. (c) Composition of plasma protein corona formed on SWCNTs [40]. Reproduced with permission. Copyright 2020, Wiley‐VCH. (d) Distribution of MWCNTs in the kidney, spleen, lung, liver, brain, and heart of a mouse model [45]. Reproduced with permission. Copyright 2015, Springer Nature. (e) Immune response induced by unfolded corona protein around MWCNTs [48]. NK: natural killer cell, DC: dendritic cell. Inset, schematic illustration, and transmission electron microscopy (TEM) images of the unfolding of AGP and IgG onto MWCNTs. Reproduced with permission. Copyright 2021, Wiley‐VCH. (f) The structure of a recombinant protein HER2Rbp, and its protein affinity and targeting efficacy after coating onto MWCNTs [31]. Reproduced with permission. Copyright 2021, Springer Nature.

CNTs are one‐dimensional seamless cylinders consisting of sp^2^‐bonded carbon atoms, which can be imagined as rolled‐up graphene sheets. Depending on the number of concentric layers, e.g., single‐ or multi‐layers, CNTs are classified as single‐walled carbon nanotubes (SWCNTs) or multi‐walled carbon nanotubes (MWCNTs), respectively. Accordingly, the diameter of SWCNTs (0.5–1.5 nm) is smaller than that of MWCNTs (>10 nm), while the specific surface area of the former (∼1300 m^2^/g) is relatively large as compared to that of the latter (100–500 m^2^/g). The lengths of SWCNTs and MWCNTs can vary from dozens of nanometers up to several micrometers and even centimeters, resulting in a very high aspect ratio (Figure 2a).

### 2.1 Effect of CNT Property on Protein Adsorption

Due to the hollow tubular structure, CNTs present a variety of binding sites for protein adsorption on both the outer surface and the inner wall [30]. Up to 60 wt.% proteins of the total mass of CNTs were quantified [31], with the smaller diameter MWCNTs capturing higher amounts of these biomolecules [32]. In an earlier proteomic study [33], more than 750 proteins were identified on MWCNTs (with diameters > 40 and 100 nm), after their incubation with lysates extracted from human cells, a representative source of the human plasma proteome. Further analysis of protein composition revealed that complement and immunoglobulin proteins were enriched in the corona layer with respect to their original abundance in biofluids [34], while serum albumin tended to decrease its presence onto CNT surface [35]. This phenomenon suggests that the interaction of proteins and CNTs is a selective process, rather than random aggregation.

Generally, proteins with a structure adaptable to CNT curvature and/or morphology are likely to have favorable interactions. For instance, fibrinogen, a hexameric glycoprotein, consisting of a linear array of three nodules in a filamentous sigmoid‐like shape, is prone to bind to SWCNTs in a lengthwise manner (Figure 2b‐i) [36]. In order to gain insight into CNT‐protein complexation, the interactions of SWCNTs with bovine fibrinogen (BFG), gamma immunoglobulin (γ‐Ig), transferrin (Tf), and bovine serum albumin (BSA) were probed [37]. The linear correlation between the order of protein adsorption rates (BFG > γ‐Ig > Tf > BSA) and the number of hydrophobic exposed amino acids in the protein molecules (88, 37, 29, and 20 for BFG, γ‐Ig, Tf, and BSA, respectively) highlighted the importance of hydrophobic interactions in driving protein corona formation onto CNTs. In addition, the correlation between the total numbers of aromatic residues, including phenylalanine, tyrosine, and tryptophan, and the amounts of adsorbed proteins, further confirms the contribution of the π–π stacking interactions between the aromatic residues and the graphitic surface of SWCNTs (Figure 2b‐ii). Besides the key role played by aliphatic and aromatic residues, the arrangement of amino acids or the hierarchical structure of proteins also contributes to form a stable corona. In fact, proteins containing high contents of α‐helix (HSP60, HSP70, and β‐actin) were found to bind more to MWCNTs, to the detriment of those rich in β‐strands, contributing less to the corona [33].

### 2.2 Effect of Protein Corona on the Biological Performance of CNTs

#### 2.2.1 Effect of Protein Corona Composition on Cellular Uptake, Biodistribution, and Toxicity

Despite its relatively low affinity, albumin still constituted the major fraction of serum protein corona coating SWCNTs, probably due to its relatively high abundance in the bulk solution. This protein can promote RAW264.7 macrophage uptake of SWCNTs via the cell surface scavenger receptors [38], resulting in a rapid clearance of the nanotubes from systemic circulation. However, in the bloodstream, due to the presence of high‐affinity proteins like fibrinogen and complement C3, the low‐affinity albumin on the surface of CNTs will be replaced by high‐affinity proteins through the Vroman effect [39]. Eventually, proteins related to coagulation and complement have been identified as the main constituents of protein corona (Figure 2c) [40]. These proteins, belonging to the family of opsonins, can also initiate the recognition of CNTs by integrins [41] and/or by complement receptors [42] on macrophages, further accelerating their removal from blood circulation. Indeed, the complement opsonization was found to enhance U937 macrophage uptake of CNTs through C1q‐mediated classical pathways [43]. This has also been demonstrated by the short blood circulation of SWCNTs in rabbits (around 1 h) [44], and extensive accumulation of MWCNTs in the lung, spleen, and liver of a mouse model (Figure 2d) [45].

In addition to dynamic protein corona evolution during the blood vessel transportation, pulmonary inhaled MWCNTs are likely to undergo corona exchange as the expression level or function of proteins in the surrounding biofluids fluctuates, aggravating the pulmonary pathologies and cancer progression. It was demonstrated that pulmonary exposure to MWCNTs coated with an allergic corona acquired from protein extracts of house dust mites (enriched with Der p 2 protein) was able to raise the expression of lactate dehydrogenase in bronchoalveolar lavage fluid and to trigger the activation of signal transducer and activator of transcription 6 in the lung [46]. These dynamic variations resulted in a higher secretion of pro‐inflammatory mediators (encoded by *Il6* and *Ccl11* genes) and a marked increment of lung inflammatory cells, which in turn exacerbated the pathological effects on allergic lung diseases, including eosinophilia, fibrosis, and mucous cell metaplasia [46]. In another long‐term (120 days) study, pulmonary inhalation of MWCNTs was shown to upregulate the expression levels of a set of proteins (i.e., VEGFA, bFGF, and COX‐2) in murine lung tissues and serum, which were highly associated with the dissemination of breast cancer to lung and other distal organs [47]. Interestingly, the expression of VEGFA and COX‐2 was highly interdependent, and the essence of the two proteins in promoting cell invasion and cancer progression was proved via protein component supplementation or gene knockout strategies.

#### 2.2.2 Structure Alteration of Proteins and Biological Consequences

Upon getting close to CNTs, the proteins tend to adjust their conformation to stabilize the CNT‐protein complexes in a thermodynamically favorable manner. As a result, the three‐dimensional conformations of proteins are likely disturbed after being adsorbed onto CNTs, leading to altered physiological functions, sometimes imposing deleterious effects on the organism.

By monitoring the conformational changes of BSA following its association with MWCNTs of different diameters, this heart‐shaped protein was revealed to undergo different degrees of unfolding as a function of MWCNT diameter. Analyzing the secondary and tertiary structural transformations, the smaller diameter MWCNTs induced greater distortion to the α‐helix, β‐sheet, and β‐turns, resulting in a higher extent of unfolding [32]. These structural perturbations likely affect the orientation of exposed epitopes, which, in turn, may shift the cellular uptake mechanism and uptake efficiency.

Similarly, the unfolding degree of 𝛼1 acid‐glycoprotein (AGP) and immunoglobulin G (IgG) was found higher for 10 nm‐diameter MWCNTs than that for larger diameter nanotubes (i.e., 30 nm) [48]. Due to the important roles of AGP and IgG in inflammation‐related pathways, their structural alternation consequently resulted in high generation of reactive oxygen species and release of major pro‐inflammatory cytokines in murine and human macrophage cell lines. In a mouse model, the structural variations of AGP and IgG selectively activated the innate and adaptive immunity, with neutrophils, natural killer cells, and CD8^+^ T cells overpopulated in the spleen (Figure 2e).

### 2.3 Strategies to Modulate Protein Corona Formation onto CNTs

Since the unintentionally adsorbed proteins may negatively affect the delivery efficacy and toxicity of CNTs, consequent works have been dedicated to modulate protein corona formation for obtaining desired biological effects. Surface modification of CNTs can turn the composition of protein corona, while the use of recombinant proteins can control the structure of adsorbed proteins.

#### 2.3.1 CNT Surface Chemistry to Turn Protein Corona Composition

Due to the hydrophobic nature of CNTs, they are often subjected to oxidation for enhanced dispersibility in a physiological environment. Interestingly, the hydroxy and carboxy groups onto MWCNTs were found to reduce the adsorption of serum proteins from 30 to 20 wt.%, likely alleviating the negative effect of unwanted protein adsorption [31]. On the other hand, MWCNTs with carboxy groups were reported to form large aggregates with fibrinogen, thereby decreasing their contacts with blood platelets to reduce platelet agglomeration and to enhance blood biocompatibility [49]. Replacing oxygenated functional groups on CNTs with hydrophilic polymers as surface coatings, the latter can shape the behavior of protein adsorption as well as the composition of protein corona on CNTs. By wrapping single‐stranded DNA around SWCNTs, this biomolecule acts as molecular recognition functionality to preferentially bind plasma proteins involved in lipid transportation, complement activation, and blood coagulation. In particular, hydrophobic interactions were found to dominate the binding of proteins at the inner layer, including clusterin, complement C3, and fibrinogen, while electrostatic interactions govern the attachment of albumin and haptoglobin in the outer corona layer [40]. For PEG‐coated SWCNTs, the abundance of major corona components (fibrinogen and IgG) reversed as the structure of PEG conformation transformed from mushroom to mushroom‐brush, which was driven by the increment of PEG grafting density [50]. Of note, the mushroom‐brush PEG was able to recruit tenfold higher amount of β‐2‐glycoprotein than the mushroom PEG, which in turn induced a higher splenic accumulation and fast renal excretion of SWCNTs.

#### 2.3.2 Recombinant Proteins to Modulate the Structure of Adsorbed Proteins

Apart from the physicochemical properties of CNTs, the molecular features of proteins, especially the polypeptide sequence and structural organization, also play important roles in mediating protein corona formation [31]. In this context, exploiting recombinant protein is a possible approach. For example, a synthetic protein was genetically engineered to bear a cationic CNT‐binding peptide at the N‐terminus and a peptide targeting the human epidermal growth factor receptor 2 (HER2) at the C‐terminus of the polypeptide chain. Based on this design, the cationic sequence was firmly attached onto the oxidized MWCNT backbone, while the opposite targeting moiety was repelled away to face outward. Since the synthetic HER2‐binding protein (HER2bp) had negligible non‐specific interaction with proteins from the incubation medium (fetal bovine serum, FBS), the targeting efficiency of this MWCNT‐protein complex toward cancer cells overexpressing HER2 receptor can be maintained (Figure 2f). The principle of this design is applicable to other CNM‐protein complexes, e.g., the desired protein conformation can be achieved by simply changing the binding peptide and targeting moiety on demand.

## 3 Graphene‐related Materials

**FIGURE 3:**
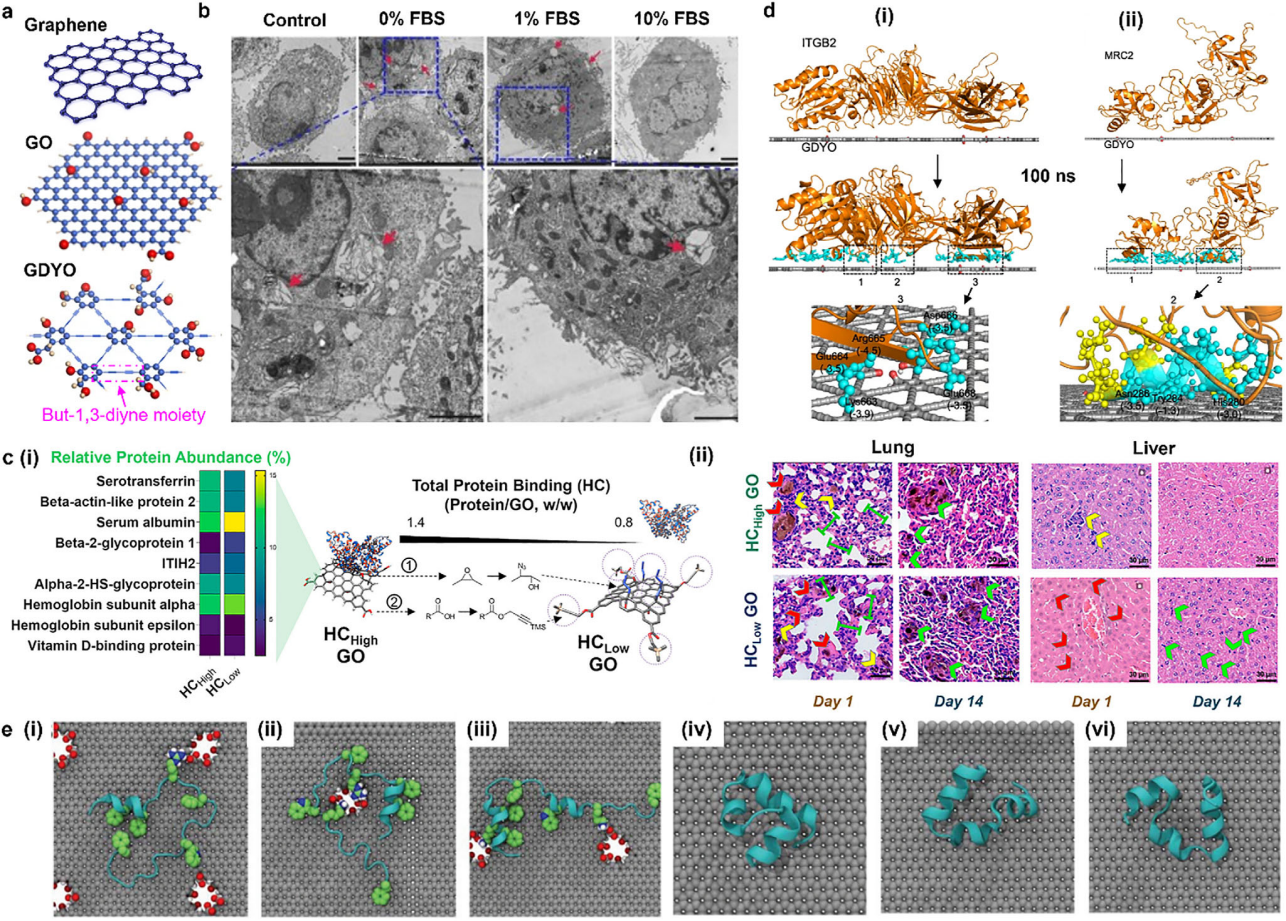
(a) Schematic representation of the structures of graphene, GO, and GDYO [70]. Carbon and oxygen atoms are shown in blue and red, respectively. Reproduced with permission. Copyright 2022, Springer Nature. (b) TEM images of A549 cells before and after treatment with GO under conditions of 0%, 1%, and 10% FBS [64]. Red arrows indicate GO nanosheets. Reproduced with permission. Copyright 2021, Elsevier. (c) The most abundant proteins detected on GO with high and low protein binding profiles (i), and lung and liver tissues of mice treated with two kinds of GO (ii) [68]. HC: hard‐bound protein corona. The arrows in (ii) are representative of damage to lung and liver tissues. Scale bar, 30 µm. Reproduced with permission. Copyright 2024, American Chemical Society. (d) MD simulation about the interactions of GDYO with ITGB2 (i) and MRC2 (ii) expressed on mutated acute myeloid leukemia [70]. Reproduced with permission. Copyright 2022, Springer Nature. (e) MD simulation about the interactions of HP35 with defective (i–iii) and intact graphene (iv–vi) [74]. Reproduced with permission. Copyright 2019, Royal Society of Chemistry.

In this section, we will mainly discuss protein corona formed on GRMs, including graphene, graphene oxide (GO), and graphdiyne oxide (GDYO) (Figure 3a). Graphene is a planar 2D nanomaterial, consisting of sp^2^‐hybridized carbon atoms arranged in a hexagonal lattice. GO is the highly oxidized form of graphene, which contains carboxy and hydroxy groups at the edges of the nanosheet as well as hydroxy group and epoxide on the basal plane. These polar surface groups not only endow GO with high colloidal stability in aqueous solutions but also serve as versatile functions for further modification with various biological molecules for medical applications. GDYO is a recently emerged graphene derivative composed of sp‐ and sp^2^‐hybridized carbon atoms, bearing but‐1,3‐diyne moiety (made of alternated single‐ and triple‐bonds) at the conjunctions of hexagonal rings. The oxygen‐containing groups in GDYO are attached either to the sp‐ or sp^2^‐carbons.

### 3.1 Effect of GRM Property on Protein Adsorption

According to molecular dynamics (MD) simulation studies, the binding affinity of proteins to CNMs is affected by their contacting surface curvatures [51], and the flat surface of graphene favors protein anchoring, leading to an enhanced adsorption capacity compared to CNTs. The inverse correlation between the curvatures of CNMs and protein affinity is responsible for a higher contact number of BSA with GRMs than with CNTs, the trend becoming more distinct as the curvature of CNTs increases [52]. In addition, the amount and species of proteins adsorbed onto GO flakes increase as the concentration of plasma proteins in the incubation medium increases, while the lateral size of GO has almost no effect on protein corona formation [53].

Due to its high concentration in serum or plasma, albumin is abundantly detected in the protein corona formed onto graphene and GO surfaces. Aside from this protein, immunoglobulins and lipoproteins are also the main components of protein corona formed on hydrophobic graphene flakes [54], presumably due to the strong π–π stacking interactions between the aromatic residues from these proteins and the sp^2^‐carbons in graphene. However, MD simulation revealed that the contact surface area of immunoglobulins with GO sheets reduces as the number of hydrophilic functions on GO increases, while the contacts of lipoproteins with the hydrophilic surface remain unaffected [55]. As a result, lipoproteins (e.g., ApoE) [55], along with other glycoproteins (e.g., alpha‐2‐HS‐glycoprotein) [56] turned out to be major fractions of protein corona coating GO.

### 3.2 Effect of Protein Corona Formation on the Biological Performance of GRMs

#### 3.2.1 Effect of Protein Corona on Cellular Uptake, Biodistribution, and Toxicity

Despite the depletion of immunoglobulins on GO surface, the presence of these proteins (e.g., IgG, IgE, IGHG, and IGKC), along with the complement C3, facilitates the recognition of GO by Fc and complement receptors on macrophages to promote subsequent phagocytosis [57], resulting in a non‐specific retention of GO in the lung, liver, and spleen. Noteworthy, GO with an IgG‐enriched corona is preferentially retained in the lung tissue, and its accumulation enhances as the administered concentration of GO increases [58]. A similar trend was observed in the biodistribution study using Rhenium‐labelled GO, where the lung accumulation still predominated after two days from the injection [59]. Aside from off‐target distribution, IgG also elicited a severe pro‐inflammatory response after two weeks’ exposure. Likewise, GO with a plasma protein corona isolated from patients with blood cancer was reported to exert inflammatory responses in breast cancer cell lines (i.e., MCF‐7 and MDA‐MB‐231) and to aggravate the cytotoxic effects. This behavior can be presumably attributed to the distinct corona pattern of a diseased population, such as a high content of complement factors or the unfolding of specific proteins (e.g., albumin and fibrinogen), which enhance the cellular uptake of GO [60, 61].

However, the protein coronas formed in FBS were shown to impede the cellular internalization of GO, and the reduction of cellular uptake was proportional to the amount of adsorbed proteins [62, 63]. As demonstrated by TEM images (Figure 3b) [64], pristine GO without FBS corona induced substantial cellular uptake by lung cancer A549 cells, while the internalization of GO was remarkably reduced under culture conditions containing 1% FBS. Noteworthily, when increasing FBS concentration to 10%, GO was rarely detected inside cells. The reduction of cellular uptake consequently alleviated the cytotoxicity of GO. As compared with the low cell viability induced by pristine GO (50%), GO coated with 1% and 10% FBS corona preserved viable cells to around 70%–80% and 100%, respectively [65]. Experiments and MD simulations demonstrated that pristine GO nanosheets can fully penetrate into the hydrophobic interior of lipid bilayers to form a sandwiched GO‐cell membrane superstructure, creating hemi‐pores in the leaflets of the bilayer membrane leading to its disruption [66]. However, the protein corona coating reduces the contact surface area of GO with the cell membrane and limits their insertion into the bilayer, consequently reducing the physical damage of the cellular membrane and cytotoxicity [67].

Aligning with the in vitro observations, it was evidenced that the quantity of protein corona onto GO influenced its biocompatibility and the immune responses in a mouse model [68]. Pristine GO and GO with both azide and alkyne functionalities were prepared to have high and low protein binding profiles, corresponding to 1.4 (HC_High_) (HC, hard‐bound protein corona) and 0.8 (HC_Low_) protein‐to‐graphene weight ratios, respectively (Figure 3c‐i). The histopathological examination of major organs (i.e., heart, liver, spleen, lung, and kidney) revealed that HC_Low_ GO induced more severe acute lung and liver injury compared to HC_High_ GO (Figure 3c‐ii). Additionally, the intravenous administration of HC_Low_ GO resulted in increased hematological toxicity and elevated levels of pro‐inflammatory cytokines. A detailed analysis of the protein corona composition indicated that the enhanced toxicity was related to nine serum proteins adsorbed on HC_High_ and HC_Low_ GO. For example, beta‐2‐glycoprotein 1 and vitamin D‐binding protein are involved in the immune response, so their enrichment onto HC_Low_ GO may activate the inflammation cascade. In the case of the liver injury, the higher contents of serum albumin, beta‐2‐glycoprotein 1, inter alpha trypsin inhibitor heavy chain H2 (ITIH2), and hemoglobin subunit alpha adsorbed onto HC_Low_ GO were positively correlated with serum aspartate aminotransferase (AST) levels, whereas the remaining proteins, enriched onto HC_High_ GO, were inversely associated with serum AST.

Owing to the presence of but‐1,3‐diyne moiety and oxygen‐containing groups, GDYO can preferentially interact with intracellular proteins and those expressed on the cell surface. For the former condition, GDYO is reported to interact with signal transducer and activator of transcription 3 (STAT3), an intracellular protein involved in the processes of tumorigenesis and immune responses [69]. The interactions of the two components are favored by structural matching, i.e., a similar distance between two adjacent C═O and/or C─OH groups in GDYO (5.5 Å) and those of the consecutive turns of the α‐helical pitch located at the N‐terminal domain of STAT3 (5.4 Å). In addition, hydrogen bonding and salt bridges further strengthen the attachment of STAT3 onto GDYO. Given the important role of STAT3 in macrophage polarization, this selective interaction allows immunomodulation within tumor microenvironment, promoting cancer immunotherapy. Indeed, such immunomodulation was not observed in macrophages with depleted gene encoding STAT3 expression, highlighting the essential contribution of GDYO‐STAT3 interaction to immunotherapy. As for the latter case, GDYO is demonstrated to bind to integrin β2 (ITGB2) and c‐type mannose receptors (MRC2) overexpressed on mutated acute myeloid leukemia [70]. MD simulation reveals that the binding is stable at specific domains within ITGB2 and MRC2, which contain abundant hydrophilic and positively charged amino acid residues, including lysine, arginine, and histidine (Figure 3d). Since ITGB2 and MRC2 are involved in the signaling pathways of cell adhesion and phagocytosis, the preferential binding between GDYO and these two corona components will promote the subsequent cellular uptake, leading to an efficient treatment against leukemia. Of note, fibrinogen and collagen facilitate the interaction of GDYO with ITGB2 and MRC2, respectively, thereby enhancing the therapeutic effect of this carbon material.

#### 3.2.2 Structure Alteration of Proteins and Biological Consequences

Owing to their high surface energy and flat basal planes, graphene nanosheets possess the strongest capacity for altering the protein structure among CNMs. For example, it was shown that the secondary structure of BSA quickly changed after packing onto graphene [71]. In addition, the degree of structural alteration, e.g., the transformation of α‐helix and β‐sheet to the β‐coil/turn structures, positively correlated with the contact surface area. For naturally occurring serum albumin, its hydrophobic residues are buried at the interior, while the hydrophilic amino acids are exposed to the surrounding aqueous environment. During the adsorption of proteins onto graphene, the hydrophobic and hydrophilic domains of this protein are reorganized to enthalpically favor the stabilization of the complex with graphene. Similar phenomena were also observed in a variety of other proteins, including IgE, myoglobin [72], and the globular domain of prion protein [73].

Noteworthy, the structural defects in graphene, produced during ion irradiation or chemical treatment processes, were found to play a critical role in protein denaturation [74]. In this simulation study, graphene nanosheets with vacancies of ∼1 nm diameter were created as a model material. The edges of these defects were designed to have twelve carbon atoms, which were conjugated to carboxy groups and hydrogen atoms in a saturated state. All‐atom MD simulation demonstrated that the defective regions serve as anchoring sites to immobilize HP35 protein. The carboxy groups on the defect edges interact with the charged residues (e.g., Lys‐7, Arg‐14, Lys‐24, and Lys‐29) at the surface of HP35 via electrostatic interactions. The strong attractive forces caused the unfolding of the protein near the anchoring sites, subsequently inducing the exposure of aromatic residues (Phe‐6, Phe‐10, Phe‐17, Trp‐23, and Phe‐35) at the core for π–π stacking interactions with the defect‐free regions in the graphene sheets. This, in turn, leads to the denaturation of the adsorbed protein, disturbing protein native functions and posing toxicity to the biological system (Figure 3e‐i–iii). On the contrary, both secondary and tertiary structures of HP35 were largely maintained on intact graphene, with limited aromatic residues (mainly the Trp‐23 and Phe‐35 in helix‐3) involved in the binding (Figure 3e‐iv–vi). These findings highlight the importance of tailoring the conformation of proteins bound to GRMs.

### 3.3 Modulating Protein Corona Formation onto GRMs

Here, we will discuss the surface modification of GRMs and the engineering of proteins for a controlled protein corona formation.

#### 3.3.1 Surface Chemistry to Turn Protein Corona Composition

The non‐specific interaction of opsonins with the RES organs spurs numerous surface modification strategies to prevent protein corona formation onto GRMs. Among them, PEGylation is the most widely used. Due to the high protein affinity of GRMs, branched PEG that can afford a denser coating is more effective than the linear version in preventing corona coating. For instance, GO modified with 4‐armed PEG was able to co‐deliver anticancer drugs cisplatin and doxorubicin into tumor sites [75], while the one functionalized with linear PEG was mainly trapped in the liver and spleen after intravenous injection [76]. Other than reducing the total amount of protein adsorption, depleting the opsonins from generating the corona layer is another strategy to optimize the organ biodistribution. For instance, polar and neutral hydroxy groups serve such kind of purpose [55]. By increasing the availability of hydroxyl groups on the graphene surface to weaken their interactions with albumin and immunoglobulins, the abundance of opsonins in the corona layer was reduced, while dysopsonins were enriched. As such, GO pre‐coated with dysopsonin protein (ApoE) exhibited prolonged blood circulation, reduced liver and spleen retention, and enhanced tumor accumulation, as compared to the pristine GO and the one pre‐adsorbed with opsonin IgE.

**FIGURE 4:**
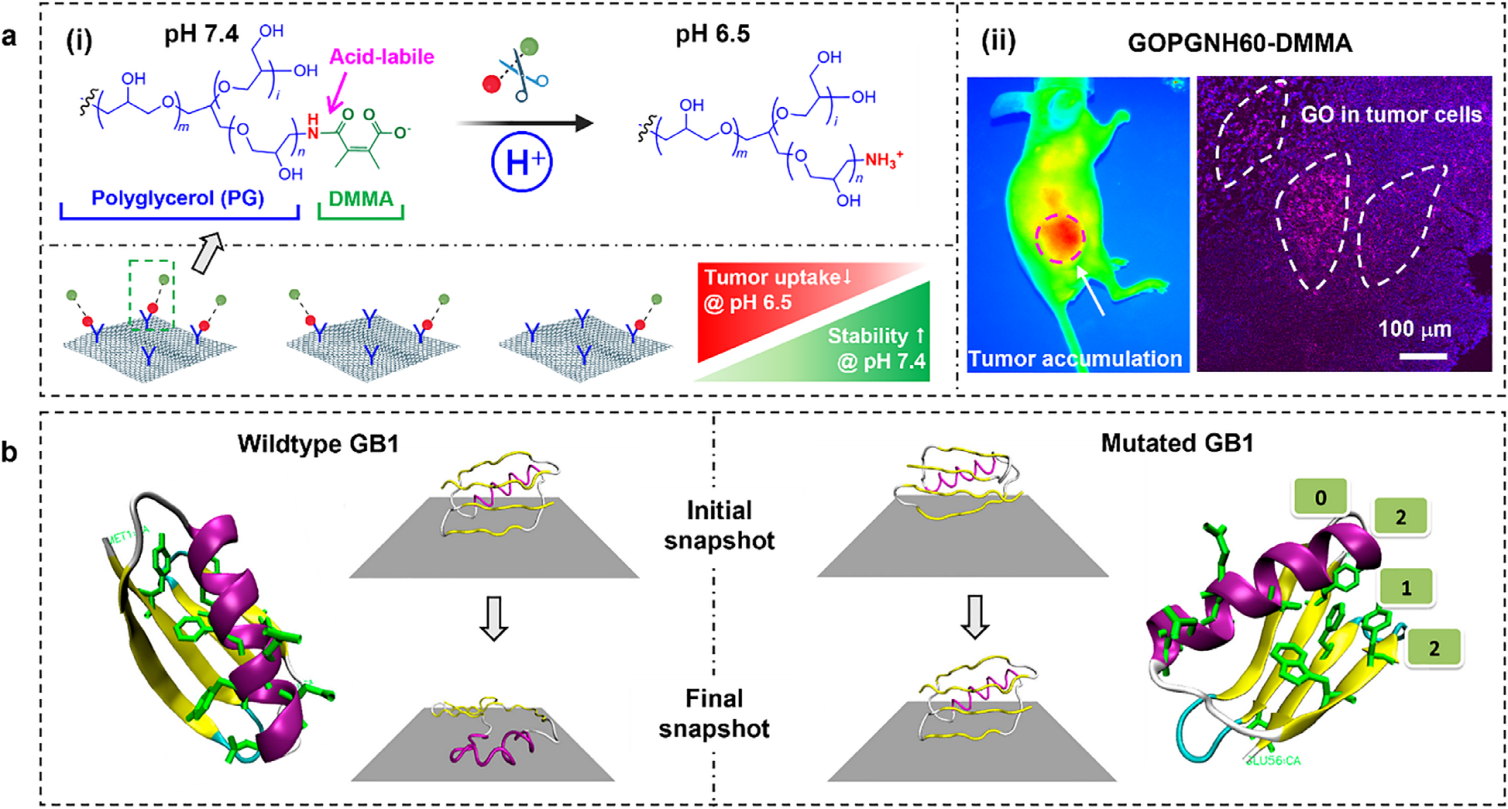
(a) Schematic representation of charge conversion of GOPGNH‐DMMA at pH 7.4 and 6.5 (i), and in vivo distribution and tumor cellular uptake of GOPGNH60‐DMMA (ii) [77]. Scale bar 100 µm. Reproduced with permission. Copyright 2025, Wiley‐VCH. (b) MD simulation of the adsorption of wildtype and mutated GB1 onto the graphene surface at initial and final snapshots. Structures of wildtype GB1 before adsorption and mutated GB1 after adsorption are given. α‐helix, β‐sheets, and aromatic residues are highlighted in purple, yellow, and green, respectively [78]. Reproduced with permission. Copyright 2019, American Chemical Society.

Nonetheless, to efficiently eradicate tumor lesions, enhanced tumor accumulation is not enough. In vivo tumor cellular internalization is a crucial prerequisite. To this end, a pH‐responsive charge‐convertible surface on poly(glycerol) (PG) grafted GO allows dynamic control in vivo protein corona formation for on‐demand tumor cellular uptake [77]. In this design, the amino groups from GOPGNH_2_ are protected by the acid‐labile dimethylmaleic anhydride (DMMA), exhibiting negative charge under physiological conditions (pH 7.4), hampering protein adsorption and non‐specific uptake. At the acidic tumor microenvironment (pH 6.5), the cleavage of DMMA induces amine exposure to promote protein adsorption and to enhance tumor cellular internalization (Figure 4a‐i). Among the different GOPGNH‐DMMA variants, those prepared from higher amine density experienced faster charge conversion to result in non‐specific accumulation, whereas those derivatized from lower amine density favored tumor accumulation. Notably, GOPGNH60‐DMMA (prepared with an amine content of 60 µmol/g) associated with enough amounts of proteins also at pH 6.5, promoting tumor cellular uptake (Figure 4a‐ii). These findings will open a new road to tailor protein corona formation for precise cancer therapy.

#### 3.3.2 Recombinant Proteins to Modulate the Structure of Adsorbed Proteins

In addition to the optimization of protein corona composition, maintaining the native structure of adsorbed proteins for proper conformational orientation is critical for cellular recognition of nanomaterials. This biological requirement was achieved by applying a protein mutation strategy, in which the IgG antibody‐binding domain of protein G (GB1) was selected as a model protein [78]. In the case of wildtype GB1, the high density of aromatic residues from the α‐helical component strongly interacted with graphene, leading to substantial conformational changes and denaturation of the entire protein. The structural disruption was reflected by the flat signals of amide I band in the region of 1500–1800 cm^−1^ in the sum frequency generation (SFG) spectrum. After understanding the structural changes of GB1 and its interaction with graphene, a redesigned GB1 with only two aromatic residues (Q32A and N35A) mutated in the α‐helical domain was synthesized. Accordingly, the π–π stacking interaction between α‐helical domain and graphene was weakened, leaving α‐helical structure standing up or tilting on graphene to retain the native conformation of mutated protein (Figure 4b). The preservation of the α‐helical structure was clearly evidenced by the increased signals of amide I band in the SFG spectra. This study can inspire how to tune the conformation of proteins via rational design.

## 4 Nanodiamonds

**FIGURE 5:**
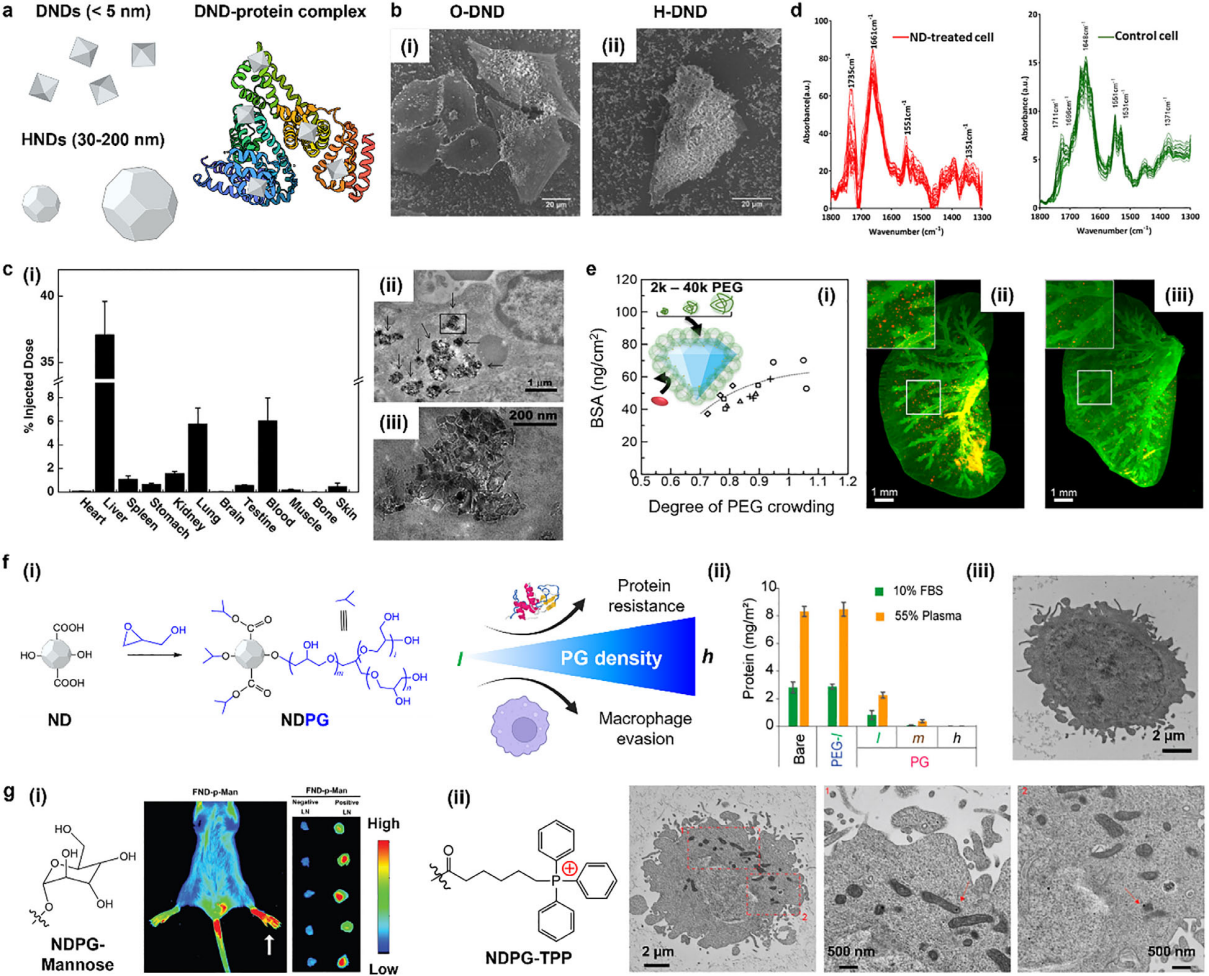
(a) Schematic representation of the structures of DNDs and HNDs, and the complexation of DNDs with large‐sized proteins. (b) SEM images of sarcoma osteogenic cells after their incubation with oxygen‐ and hydrogen‐terminated DNDs (O‐DND (i) and H‐DND (ii), respectively) [87]. Reproduced with permission. Copyright 2020, Elsevier. (c) Biodistribution of pristine HNDs in major organs of the mouse model (i), and TEM images of liver tissues after 28‐day treatment (ii, iii) [94]. Reproduced with permission. Copyright 2009, Elsevier. (d) Infrared spectra of intracellular proteins with and without DND treatment. Reproduced with permission. Copyright 2016, American Chemical Society [98]. (e) Effect of PEG crowding on BSA adsorption (i), and the accumulation of HND‐COOH (ii) and HND‐PEG20k (iii) in lung tissue of mice [99]. Reproduced with permission. Copyright 2024, American Chemical Society. (f) (i) Schematic illustration of the synthetic process of NDPG, and density‐dependence of PG on protein resistance and macrophage evasion. (ii) Proteins detected on HND (size: 100 nm) grafted with PEG and PG in varying densities. *l*, *m*, and *h* represent low, medium, and high contents of polymer grafting. (iii) U937 macrophage uptake of HNDPG‐*h* [90]. Reproduced with permission. Copyright 2020, American Chemical Society. (g) NDPG derivatized with mannose (i) [109] and TPP (ii) [111] at appropriate densities for tumor draining lymph node imaging and mitochondria targeting, respectively. Reproduced with permission. Copyright 2022, Wiley‐VCH. Copyright 2021, American Chemical Society.

NDs typically consist of a sp^3^‐hybridized lattice carbon core and a graphitic shell with various surface groups [79]. Depending on the preparation methods, the size of NDs can vary from a few to hundreds of nanometers (Figure 5a). For instance, NDs with diameters less than 5 nm can be generated in large quantities by detonating carbon‐rich explosives in an inert environment [80]. In contrast, NDs with relatively larger sizes (30–200 nm) can be produced from bulk diamond microcrystals via high‐pressure/high‐temperature and laser ablation [81] approaches or from ultrasmall diamond particles through chemical vapor deposition [82] methods. Aside from the size diversity, NDs can be further modified with a variety of functional molecules at the outer surface to treat diseases [83] or modified with vacancy centers at the inner lattice core for imaging [84] and biosensing [85] applications. As NDs synthesized from detonation (DNDs) and high‐pressure/high‐temperature (HNDs) methods are prevailing in current research, we will mainly summarize the protein corona studies on these two types of NDs.

### 4.1 Effect of ND Property on Protein Adsorption

The ultrasmall size of DNDs is a unique characteristic compared to other CNMs. In a recent study about the interaction of small nanoparticles with proteins, when the size of ENMs is comparable to or smaller than the size of proteins (e.g., 5–20 nm), these particles tend to bind to the protein surface to form a protein complex, rather than being coated by a layer of corona [86]. A schematic illustration of the association of DNDs with large‐sized proteins is present in Figure 5a. By investigating the interactions of FBS proteins with DNDs of different surface terminations, it was demonstrated that the binding between the two components was mainly mediated by electrostatic interactions [87]. Hydrogen‐terminated DNDs (positively charged) were preferentially associated with proteins with an isoelectric point (pI) < 7 (e.g., proteins exhibiting a negative charge at physiological pH), while proteins with pI >7 were mostly detected on the surface of oxygen‐terminated DNDs (negatively charged). However, a further study using BSA (pI: 4.8) and lysozyme (LYS, pI: 11) as model proteins reveals that the interactions of charged DNDs with these proteins are not simply driven by the electrostatic attraction and/or repulsion forces. Using a quartz crystal microbalance‐based assay, both BSA and LYS were found to adsorb onto DNDs of different surface charge in comparable amounts [88]. This behavior was explained by the fact that charged DNDs would attract counterions from the buffer solution to form a water hydration layer, which consequently created an extremely high or low pH environment near the DND surface. As a result, the charge distribution of specific amino acid residues in proteins was modified, thus, the interactions between proteins and DNDs were reshaped.

Different from DNDs, the surface chemistry of HNDs has a negligible effect on protein corona formation. Exploring HNDs bearing amines and carboxylate functionalities, the top thirty most abundant proteins of FBS corona, which constituted almost 95% of all detected proteins, were similar in terms of size (similar molecular weight), abundance, and biological functions [89]. In addition, the number of adsorbed proteins was found to be independent of HND size, when normalized by the total surface area of the particles. The proteomics analysis of plasma corona formed on 100 nm‐sized HNDs revealed that immunoglobulin, coagulation factors, and acute phase family proteins enriched their abundance onto HND surface, while the quantity of serum albumin decreased [90].

### 4.2 Protein Corona Redefining the Biological Performances of NDs

#### 4.2.1 Effect of Protein Corona on Cytotoxicity, Immune Response, and Biodistribution of NDs

The effects of adsorbed proteins on the toxicity of NDs vary as the surface chemistry of NDs and the incubation conditions differ. Concerning NDs with different surface chemistry, the associated proteins are prone to alleviate the toxicity of pristine and negatively charged NDs, opposite to positively charged NDs still exerting toxic effects. For instance, the FBS proteins reduced the toxicity of oxygen‐terminated DNDs toward sarcoma osteogenic cells, while hydrogen‐terminated DNDs remained toxic in the presence of FBS proteins [87]. In the scanning electron microscopy (SEM) images, oxygen‐terminated DNDs only partially cover the surface of sarcoma osteogenic cells (Figure 5b‐i), whereas hydrogen‐terminated DNDs form large aggregates with FBS proteins to fully cover the cell surface (Figure 5b‐ii). This may potentially restrict the natural movement of cells to elicit toxicity. On the other hand, the toxicity dependence of DNDs was explored in terms of protein corona composition and cell types [91]. In the case of rat hepatoma cells, both pristine and aminated‐DNDs coated with BSA were less toxic than those covered by fibronectin. The higher toxicity of the latter arises from the enhanced uptake of DNDs in the presence of fibronectin, which consequently induces high intracellular agglomeration of DNDs to compromise cell viability. However, for RAW 264.7 macrophages, both types of DNDs induced substantial cell death, regardless of the composition of protein corona (i.e., BSA or fibronectin). This can be explained by the high expression of scavenger and integrin receptors on RAW 264.7 macrophages to facilitate the phagocytosis of DNDs with both types of coronas, resulting in higher cytotoxic effects.

Apart from cytotoxicity modulation, the interaction of NDs with immune‐related proteins may also activate an immune response and further alter their in vivo distribution. For example, the binding of HNDs to C1q, the recognition unit of the first complement protein complex C1, was reported to promote the engulfment of NDs by THP‐1‐derived macrophages via the classical complement pathway. This consequently triggers the complement cascade and enhances the secretion of the cytokines IL‐6 and IL‐8 [92]. Similarly, IgG conjugated onto the surface of fluorescent NDs effectively boosted the production of TNF‐α and IFN‐γ in monocytes and natural killer cells, activating the innate immune response [93]. On the other hand, the enrichment of complement and immunoglobulin proteins on the HND surface upregulated phagocytosis by U937 macrophages [90]. This opsonization process is considered the main factor initiating the non‐specific retention of NDs in RES organs. Actually, shortly after intravenous administration, HNDs were prominently accumulated in the liver of mice (Figure 5c‐i), and around 60% of injected NDs were persistent in this organ over 28 days (Figure 5c‐ii–iii) [94]. The tissue level examination revealed that most HNDs were captured by liver macrophage, i.e., Kupffer cells, but not hepatocytes.

#### 4.2.2 Structure Alteration of Proteins and Biological Consequences

Since the interaction between proteins and ENMs weakens as the curvature of ENMs increases, DNDs with high surface curvature alter the protein structures to a lower extent. For instance, BSA preserved most of its structural features after attaching onto DNDs, e.g., only slight perturbances of α‐helix and/or β‐sheet content, and the folding of polypeptide chains was observed [95]. Similarly, the globular protein myoglobin maintained its structural integrity after forming a monolayer coverage on the surface of DNDs [96]. However, due to the presence of graphitic layers and charged species on the DND surface, structural distortions could occur in some specific proteins. In the case of insulin (Ins) with low molecular weight (e.g., 5.8 kDa), tightly bound Ins‐multimers were formed on the surface of positively charged DNDs. This would potentially result in a significant increment of intermolecular β‐sheet via strong protein‐protein attractions among Ins molecules [96]. The highly positively charged LYS, on the other hand, was reported to form multilayers onto DNDs accompanied by the structural transition from α‐helix to β‐sheet and amorphous forms [88]. These secondary structural transformations, in turn, impaired the enzymatic activity of LYS, which was not observed in HNDs with sizes over 100 nm [97]. To further explore the structural disturbance of proteins on their biological functionalities, the conformational changes of intracellular proteins were probed after the internalization of DNDs into hepatoma cells [98]. By coupling atomic force microscopy with infrared spectroscopy, the subtle changes of the cellular protein structure were detected at nanoscale resolution. In addition to the common features of secondary structural alterations, it was revealed that the ratio of amide I to amide II peak intensity was enhanced (Figure 5d), indicating the occurrence of a DND‐induced oxidative stress inside cells. Since intracellular protein conformations are highly related to the cellular response and functional outcomes of NDs inside the body, understanding the protein adsorption mechanism is crucial for identifying the optimal conditions for the safe use of NDs in biomedical applications.

### 4.3 Strategies for Modulating Protein Corona Formation onto NDs

In this section, strategies for modifying NDs with hydrophilic polymers and their derivatives to tailor protein corona formation are introduced. Particularly, the use of quantitative surface chemistry to control the density array and/or the structural display of functional groups onto the ND surface for desired biological outcomes is discussed.

#### 4.3.1 Reducing Overall Protein Adsorption by Polymer Coating

To mitigate the unwanted effects of protein corona formation, NDs are often modified with hydrophilic and electrically neutral polymers for anti‐fouling. As dense surface coverage is essential for preventing protein adsorption, PEG with varying molecular weights was examined for the crowding effect on HNDs [99]. It was revealed that PEG with a larger molecular weight afforded a higher degree of crowding onto HND surface, and thus a better prevention effect toward protein adsorption (Figure 5e‐i). In a mouse model, HNDs grafted with PEG of 20 kDa exhibited significantly low accumulation in lung tissues after intravenous injection (Figure 5e‐ii,iii), likely resulting from the higher degree of PEG reduced interactions of HND‐PEG with blood components. Although effective, the introduction of PEG into a physiological environment may disturb the host's metabolism and can induce immune responses. For instance, the development of anti‐PEG antibodies, especially anti‐PEG IgM and IgG following the initial administration of PEGylated ENMs, has been extensively reported to adversely affect their blood circulation [100, 101]. Indeed, upon subsequent injections, these antibodies bind to PEG chains on the surface of ENMs, initiating the complement activation and opsonization, which in turn accelerates the blood clearance of ENMs via hepatic and splenic macrophage uptake [102]. This process eventually leads to decreased delivery efficacy and may elicit potential immunotoxicity, urging the exploration of more biocompatible polymers for the safe application of NDs in particular, but generally to avoid the same drawbacks for all classes of ENMs. In a comparison study about the anti‐fouling effects of zwitterionic moieties, efficient inhibition of protein corona formation onto DNDs was achieved with the functionalization of tetra(ethylene glycol) linker bearing three zwitterionic head groups [103]. However, to prepare such a well‐organized hierarchical architecture onto DND surface, modification of DNDs with anchor groups and pre‐synthesis of splitter molecules were required for the immobilization of tetra(ethylene glycol) and for the control of the number of zwitterionic moieties, respectively. This multi‐step functionalization procedure limits its practical application.

In pursuit of a facile functionalization approach to prevent protein corona formation onto NDs, our group has explored the potential of PG grafting, which can be easily achieved by reacting NDs with glycidol in one pot [104]. In addition, the grafting density or the thickness of the PG layer can be fine‐tuned by controlling the reaction conditions, including reaction temperature [90] and the mass ratio of glycidol to NDs [105], enabling the quantitative estimation of the anti‐fouling efficacy of PG against its functionalization degree. Through a systematic investigation, we revealed that the higher PG grafting content corresponded to a higher efficiency of HNDPG to reduce protein corona formation and macrophage uptake (Figure 5f–i). In particular, 30 wt.% PG coating prevented corona formation onto HNDs almost completely (Figure 5f‐ii), regardless of the particle size and protein source, leading to the complete evasion of macrophage uptake (Figure 5f‐iii) [90]. As a result, the conjugates of HNDPG were able to moderate rapid sequestration by liver and spleen in the mouse model, preferentially accumulating at the tumor site for precise tumor imaging [106]. Indeed, the superiority of PG coating to evade RES trapping was also evidenced in other ENMs such as boron carbide [107] and boron nitride nanoparticles [108].

#### 4.3.2 Tailoring Corona Formation onto NDPG Derivatives for Targeted Delivery

In addition to tumor‐specific accumulation, sufficient internalization into target cells or subcellular organelles is another crucial factor. The large amounts of hydroxy groups in PG layer not only endow protein resistivity to NDPG but also serve as versatile platforms for functional programming, permitting tailored corona formation for targeted delivery. For instance, a polyvalent array of mannose on PG grafted fluorescent NDs selectively targeted macrophages in sentinel lymph nodes, allowing a clear visualization of tumor‐draining lymph nodes (Figure 5g–i) [109]. This is promising to advance the endoscopic/robotic guided tumor surgery. In another study about the tumor cellular uptake of HNDPG derivatized with charged functional groups [110], it was demonstrated that carboxylate and sulfate at lower density exhibited no affinity to proteins, inducing negligible uptake. In contrast, sulfate at higher density attracted vitronectin to suppress macropinocytosis and clathrin‐mediated endocytosis, while ammonium adsorbed proteins of the hyaluronan binding family, including inter‐alpha trypsin inhibitor heavy chains H2 and H3, and alpha‐2‐macroglobulin to enhance macropinocytosis and caveolae‐mediated endocytosis. As for subcellular targeting, triphenylphosphonium (TPP), a mitochondrial targeting moiety, was conjugated onto HNDPG at low and high densities to modulate protein corona formation [111]. The investigation revealed that TPP at lower density attracted a small number of proteins to preserve its mitochondrial targeting ability (Figure 5g‐ii), whereas a large amount of proteins on higher density TPP diminished the targeting specificity by trapping NDs inside the endosomal and lysosomal compartments.

## 5 Carbon Dots

**FIGURE 6:**
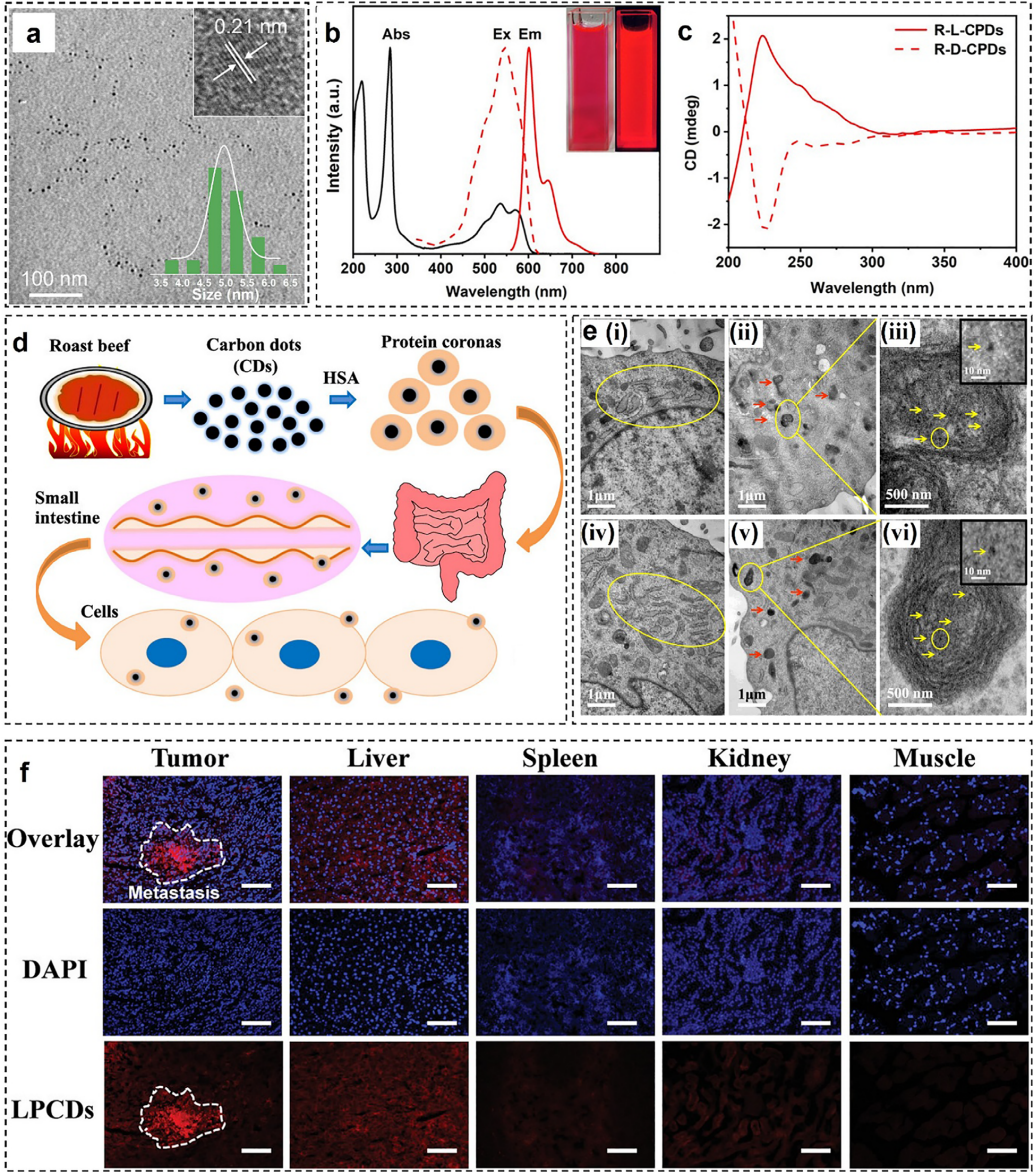
(a) TEM images of CDs. Reproduced with permission [113]. Copyright 2025, Elsevier. (b) UV–vis and fluorescence spectra of CDs. Reproduced with permission [118]. Copyright 2021, Wiley‐VCH. (c) Circular dichroism spectra of chiral CDs. Reproduced with permission [118]. Copyright 2021, Wiley‐VCH. (d) Schematic illustration of protein corona formation of CDs derived from roast beef. Reproduced with permission [131]. Copyright 2020, American Chemical Society. (e) TEM images of kidney cells grown in a serum‐free culture medium with CDs and HSA‐CDs. Reproduced with permission [131]. Copyright 2020, American Chemical Society. (f) Ex vivo tissue fluorescence microscopy of major organs collected from mice bearing MDA‐MB‐231 tumor. Reproduced with permission [137]. Copyright 2022, Wiley‐VCH.

CDs are a class of carbon nanomaterials, with diameters smaller than 10 nm (Figure 6a) [112, 113, 114]. Depending on their morphology, size, and crystallinity, CDs can be classified as graphene quantum dots (GQDs), carbon quantum dots (CQDs), carbonized polymer dots (CPDs), and carbon nanodots (CNDs) [115, 116, 117]. Compared with other CNMs, the most attractive property of CDs for biomedical applications is their highly tunable fluorescence, which can be extended from UV to near‐infrared emission (Figure 6b), facilitating bioimaging of cells in vitro and organs in vivo [118, 119]. In addition, there are abundant precursors available for the synthesis of CDs, ranging from small molecules to large‐sized graphitic systems, or biowaste [120, 121]. The richness in precursor choices enables the low‐cost synthesis of CDs with tunable size and optical properties. In the context of protein corona formation and modulation, CDs with different chirality using different enantiomers, which could induce specific interactions with proteins were developed [122].

### 5.1 Effect of CD Property on Protein Adsorption

The ultrasmall size of CDs may also promote the formation of nano‐protein complexes, as in the case of DNDs. In addition, the size of CDs was found to affect the protein association. For example, the adsorption process of villin headpiece on GQDs surface was investigated by all‐atom MD simulations [123]. With the increase of GQD size, the amount and binding strength of adsorbed protein residues increase, and sequentially influence the structural change of adsorbed protein. In addition, aromatic residues and residues with alkyl side chains have a high probability of strongly binding to the GQD surface through the π–π stacking interaction and hydrophobic interaction.

Generally, CDs possess abundant surface functional groups with distinct surface charges. As reported for iron oxide and gold nanoparticles, positively charged nanoparticles consistently attract higher quantities of proteins than negatively charged ones [124, 125]. In this context, the effects of the surface charge of CDs on the interaction between CDs and proteins were investigated. In one study, negatively charged PEG‐CDs and positively charged polyetherimide‐CDs (PEI‐CDs) were prepared through passivation of bare CDs using PEG and PEI [126]. The association of these two CDs with human serum albumin (HSA) in physiological conditions was dominated by electrostatic forces between HSA and PEI‐CDs, while the association of PEG‐CDs to HSA initiated by hydrophobic and van der Waals forces. In addition, it was found that PEG‐CDs did not induce perturbations of protein secondary structure. However, high concentrations of PEI‐CDs could induce conformational changes of HSA, especially the α‐helical domains.

As chirality (e.g., left‐ and right‐handedness) is ubiquitous in biological systems, chiral CDs provide a means for selective interactions with chiral structures in proteins and cells [127]. In recent years, the chirality of CDs has attracted much attention (Figure 6c). The influence of the chirality of CDs on their interaction with proteins was also studied [122]. For example, the interaction of achiral and chiral CNDs with HSA, alpha microglobulin, and transferrin was evaluated. These proteins only weakly bind to both achiral and chiral CNDs in comparison to metal nanoparticles, only HSA forming a clear protein corona. More importantly, in the case of HSA, significantly better binding of *R*‐CNDs than *S*‐CNDs and achiral CNDs was found. In the subsequent cellular uptake assay on THP‐1 derived macrophages, *S*‐CNDs induced substantially higher uptake than *R*‐CNDs, likely attributed to a lower coverage by HSA, favoring a high association with the cellular membranes.

### 5.2 Effect of Protein Corona on the Biological Performance of CDs

#### 5.2.1 Protein Corona Composition on Uptake and Distribution

Similar to other CNMs, the formation of protein corona also affects the uptake of CDs. In a recent work, the uptake of four CDs with different ζ‐potential values and/or density of charged functional groups on THP‐1‐derived macrophages was studied [128]. Among these CDs, the one exhibiting the highest ζ‐potential and charge density showed the highest uptake, while CDs possessing the lowest ζ‐potential and charge density exhibited the lowest uptake. In the analysis of protein corona, it was found that CDs with higher charge density were associated with proteins involved in the upregulation of phagocytosis (e.g., adiponectin and fibulin). In contrast, the CDs with lowest charge density tend to attract hemoglobin to impede the cellular internalization. This result indicates that not all cationic CDs are potently internalized by human macrophages, and not only the ζ‐potential value but also the charge density matters in determining protein corona composition and cellular uptake. Another work attempted to elucidate how protein corona alters the cellular uptake of CDs upon surface functionalization by glutathione (GSH) and folic acid (FA) [129]. The behavior of two different cell lines was compared. In the case of cancer cell lines, there was a significant amount of internalization of both bare CDs and GSH‐FA‐functionalized CDs without plasma protein coating. However, after pre‐coating with plasma proteins, bare CDs show reduced uptake while GSH‐FA‐functionalized CDs showed a significant enhancement in cellular internalization. A similar trend was also observed in the macrophage cell line. Interestingly, the extent of cellular uptake in macrophages after plasma protein coating was significantly less in comparison to both particles without plasma protein coating. This suggests that the pre‐coating of plasma protein corona may confer a stealth effect to CDs to avoid macrophage uptake.

The effects of protein corona on the biodistribution and subcellular localization of CDs were also analyzed (Figure 6d). Using a mouse model, CDs were orally administered to assess their biodistribution [130]. The presence of CDs in the stomach and intestine reached the maximum concentration after 2 h of administration and gradually decreased with time. Their presence in the kidney and testis slightly increased until 6 h, while the amounts of CDs in the liver, spleen, lungs, and heart were small. These results indicate that CDs can be absorbed by the digestive system and delivered throughout the body. In addition, in vitro experiments indicated that CDs were coated by a corona made of pepsin, leading to a reduction of the activity of this enzyme, consequently affecting its digestion capacity. In a subcellular localization study, rat kidney cells were incubated with either CDs or CDs with an HSA corona (Figure 6e) [131]. Although both materials were mainly localized in the lysosomes, bare CDs induced significant endoplasmic reticulum swelling as compared to HSA‐coated CDs. This suggests that pre‐coating with HSA alleviates the adverse effects on endoplasmic reticulum. In the case of bare CDs without pre‐coating, the strong binding between intracellular proteins and CDs led to excessive protein retention, resulting in a reduced protein secretory activity. As the endoplasmic reticulum is the main subcellular compartment for protein synthesis, its homeostasis impairment may lead to protein misfolding, potentially affecting the functions of the newly synthesized proteins and the physiological state of the cells.

#### 5.2.2 Structure Alteration of Proteins and Biological Consequences

CDs are able to form a ground‐state complex with glucose oxidase (GOx), primarily stabilized by hydrogen bonds and van der Waals forces involving amino acid residues in GOx's active site [132]. This binding leads to a conformational change (e.g., reduction in α‐helix content) and reduces the thermal stability of GOx, slightly inhibiting its enzymatic activity and demonstrating a competitive inhibition effect. CDs with near infrared emission were found to attenuate the GOx's capacity to produce H_2_O_2_ in HeLa cells, mitigating the enzyme‐induced cytotoxicity and cellular damage. In another report, gelatin‐derived CQDs were synthesized to evaluate their interaction with beta‐lactoglobulin (BLG), a model transporter protein [133]. Exposure to CQDs resulted in the disruption of BLG secondary and tertiary structural elements, transforming isolated helices into coiled‐coils and increasing β‐sheet content. More importantly, it was demonstrated that the interaction of BLG with gelatin‐derived CQDs adversely affects protein‐retinol ligand binding, potentially causing disruption in protein function.

### 5.3 Modulating Protein Corona Formation on CDs

#### 5.3.1 CD Surface Chemistry to Tune Protein Corona Composition

As mentioned above, the surface charge of CDs plays an important role in the formation of protein corona. The composition of the CD protein corona can be adjusted through surface chemistry. In a recent work, the changes in the CD protein corona by four cationic CDs exhibiting protonatable or non‐protonatable amine groups at their surface were investigated [128]. CD surface charge influences protein adsorption by modulating the electrostatic interactions, with higher charges favoring the binding of negatively charged, hydrophilic proteins, while lower charges promote the adsorption of smaller and more hydrophobic or positively charged proteins. In another work, five CDs with different alkyl chain lengths were prepared by using 1‐alkyl‐3‐methylimidazolium dicyanamide (alkyl chains corresponding to ethyl, butyl, hexyl, octyl, decyl) as precursors [134]. Their interaction with proteins was investigated using BSA as a model. The interaction capability of these CDs was influenced by the chain length and the presence of imidazolium moieties. The interactions between these CDs with BSA were too weak to cause a clear conformational change of the secondary structure of the protein, while the formation of the corona significantly attenuated the cytotoxicity of CDs. Despite the promises offered by surface functionalization in corona modulation, this approach may become ineffective when the intrinsic properties of CDs dominate their interactions with the proteins. For instance, the high curvature of CPDs and CNDs may restrict their interaction with rigid proteins, such as ApoE [135], and the consequent weak affinity between the two components cannot be readily enhanced through surface modification. Since ApoE is known to play an important role in the low‐density lipoprotein receptor‐mediated cellular uptake [136], this limitation likely impedes the internalization of CDs by cells expressing the corresponding receptors.

#### 5.3.2 Customized Biomimetic Protein Corona Formation

Recently, customization of protein corona onto CD surfaces can improve their biomedical performance and open new opportunities in the construction of targeted delivery systems (Figure 6f) [137]. Pheophytin carbon dots (PCDs), prepared from natural chlorophyll were enabled with a lipoid surface that enhanced their affinity for apolipoproteins, for targeting cancer cells with high lipid demands. These PCDs were post‐modified with a lipid‐PEG (LPCDs) and incubated with mouse plasma to form a protein corona. Fifty‐seven types of proteins were detected in the composition of this corona. Uptake and exocytosis are two important indicators of deep tissue penetration via transcytosis. The uptake of LPCDs by breast cancer cells in FBS‐contained medium was then examined by flow cytometry, and exocytosis of LPCDs was evidenced, quite fast and effective, as only 10% of LPCDs were retained in the cells. This rapid cellular uptake and exocytosis process found in LPCDs enabled their enhanced tumor penetration profiles. When cultured with FBS‐free medium, LPCDs could actively absorb endogenous apolipoproteins secreted by the cells into the protein corona to enhance their uptake in breast cancer cells. The protein corona‐coated LPCDs displayed enhanced affinity and sensitive detection for triple‐negative breast cancers. In terms of safety, the body weight measurements suggested no signs of toxicity up to three weeks with no observable damage to major organs by LPCDs, indicating that the protein corona does not compromise their safety. This transformation of a passive material into an active system may provide guidelines for developing facile and precise protein corona‐targeted delivery systems for clinical uses.

## 6 Conclusion and Perspectives

CNMs hold tremendous promise for tailored interaction with biological systems. In particular, the superiority of CNMs over other inorganic ENMs (e.g., gold or iron oxide nanomaterials) to recruit specific protein adsorption (see details of meta‐analysis in Tables S1–S6), makes them more attractive for biomedical applications. For instance, the enrichment of ApoE on CNMs (e.g., graphene and NDs) will not only shield RES recognition to enhance the delivery efficacy to tumor tissues, but also facilitate the transportation of therapeutic molecules for the treatment of brain and cardiovascular diseases [138, 139]. A thorough understanding of the interactions between CNMs and proteins is critical for turning the unpredictable protein adsorption toward controlled corona formation. In this review, we summarized 1) the effects of topological features of sp, sp^2^, and sp^3^ carbon atom hybridizations along with the surface chemistry of CNMs on protein adsorption and structural organization, 2) the effects of protein corona on the biological outcomes of CNMs, including cellular uptake, in vivo distribution, therapeutic efficiency, and toxicity, and 3) the strategies to reshape protein corona formation onto CNMs, including surface modification of CNMs to turn corona composition and the protein engineering approach to control the conformational changes of adsorbed proteins. To better delineate the relationships between CNM property and conformation of corona proteins, the effects of unique CNM character on structural alteration of proteins, and consequent biological outcomes are summarized in Table S7. This comprehensive knowledge will provide insights for customizing unique CNM‐protein interactions toward specific requirements.

Traditional nano‐biointeraction studies usually proceed in the following sequence: 1) preparation of CNMs or proteins according to structure‐activity correlations revealed from reported studies, 2) characterization of the materials and analysis of the protein corona formed on CNMs, and 3) application of the CNM‐protein complexes to cells or animal models to evaluate their performance. The final results give feedback about whether the design was successful or not. If it fails to meet the requirement, additional trial‐and‐error efforts will be necessary to optimize the design, making the current approach a laborious and time‐consuming task.

**FIGURE 7:**
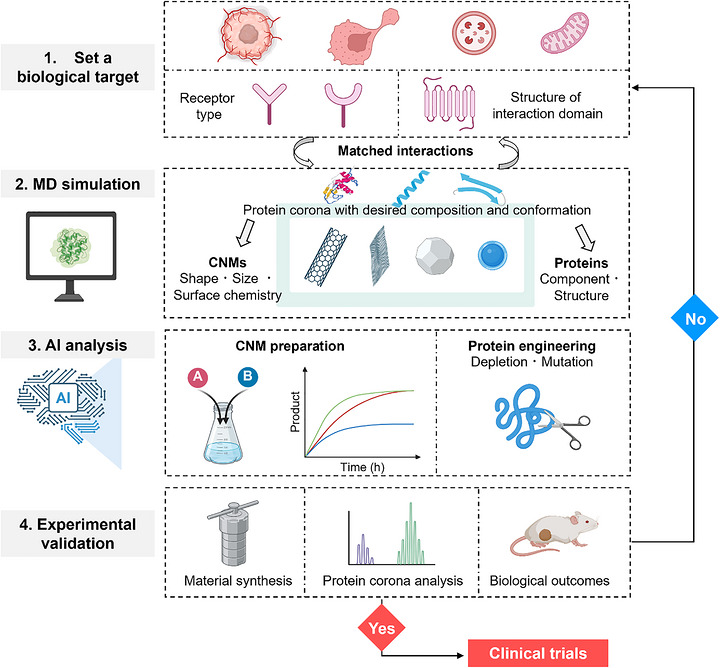
Flowchart of information‐assisted CNM preparation and protein engineering toward tailored corona formation.

As we have evidenced how MD simulations deepen our interpretation of CNM‐protein interactions at the atomic levels and drive the protein design, we suggest employing such powerful tools to foresee the interaction patterns of newly designed CNMs and proteins. Meanwhile, artificial intelligence (AI), especially machine learning (ML), can use computer algorithms to build structure/composition‐property relationships from the huge amount of available data to advance material development [140]. Advances in ML‐assisted nanoparticle synthesis have greatly accelerated the identification of the most appropriate recipe for the generation of nanomaterials with distinct properties [141]. By taking advantage of these state‐of‐the‐art approaches, we propose a forward‐thinking strategy that combines theoretical prediction and experimental validation to turn the protein corona formation on CNMs (Figure 7). The procedures of the proposed strategy should adopt the following recommendations:

1. Set the goal of biological targets, e.g., a specific organ, cell, or subcellular organelle. Identify the possible interaction sites on these targets (the type of receptors and the structure of the interaction domains) and use this information to predict the composition and conformation of corona proteins onto CNMs, ensuring that CNM‐protein complexes have matched the interactions with these biological targets.
2. Use the desired protein corona pattern on CNMs as input for MD simulation to predict the requested properties of CNMs and proteins. Select an appropriate MD simulation program to analyze the interactions of CNMs and proteins. Figure out the specific properties of CNMs (size, shape, and surface chemistry) and proteins (component and structure) that can generate desirable protein composition and conformation.
3. Use the information output from MD simulations to guide AI prediction, optimizing the conditions for CNM synthesis and protein engineering. Based on correlations of experimental parameters (type of solvent, concentration of the reactant, and reaction temperature) and CNM property (size, shape, and surface chemistry), the computer algorithms will identify the most effective combination of reagents and reaction conditions for CNM synthesis. Those for protein engineering, e.g., gene editing to exclude and/or to mutate a specific protein component, will be optimized using similar procedures.
4. Perform experiments for material preparation and protein engineering, investigate the CNM‐protein interactions, and conduct experiments to validate the therapeutic outcomes both in vitro and in vivo.
5. Those identified as positive from experimental validation could progress toward clinical trials, while negative results will go back to the feedback loop for optimization.

By efficiently organizing theoretical prediction and experimental validation, we should aim to reduce trial‐and‐error efforts in tailoring protein corona formation onto CNMs for desired functions in cancer nanomedicine. However, we should be aware that deliberate corona engineering still needs to address the reproducibility issue that may arise from different conditions of corona formation and harvesting, as well as the heterogeneity of CNM properties. For instance, substantial disparities exist in the composition of coronas formed from FBS, human serum, and human plasma. To reduce such biological variability, future studies are suggested to employ pooled biological samples or standardized commercial preparations as protein sources. Regarding the procedures of protein corona harvesting, conventional centrifugation‐based isolation methods may strip off the weakly bound proteins or perturb protein structures, posing risks of misrepresenting corona profiles. Therefore, developing gentler isolation methods will be critical to preserve the composition and structure of recovered corona. In addition, the heterogeneity of CNM properties, which include differences in particle size, morphology, and surface functionalization, may induce alterations of the conformation of the adsorbed proteins. This should be minimized through a fine control of the CNM structure during their synthesis and a rigorous physicochemical characterization.

Coupling this information‐assisted design framework with thorough, regulation‐focused characterization, scalable manufacturing, and batch‐to‐batch quality control will be key to moving innovative CNMs from bench discoveries into clinical applications.

## Conflicts of Interest

The authors declare no conflict of interest.

## Supporting information




**Supporting File**: adma73076‐sup‐0001‐SuppMat.docx.
