# Supplementary material for: Turning Unpredictable Biomolecule Adsorption to Controlled Corona Formation: Focus on Carbon Nanomaterials
Source: Adv Mater. 2026 Apr 18;38(46):e23328. doi: 10.1002/adma.202523328 (PMC13486267; doi:10.1002/adma.202523328)
Supplement: Supplementary file 1 [file ADMA-38-e23328-s001.pdf]

Turning unpredictable biomolecule adsorption to controlled corona formation: focus on carbon nanomaterials

Yajuan Zou,[1] Yalei Hu,[2] Jie Yu,[3] Naoki Komatsu,[3,] Yuta Nishina,[1,]* Alberto Bianco[1,2,]**

Supporting Information

Meta-analysis of protein corona composition on CNMs versus metal nanoparticles

Protocol. The meta-analysis was conducted for the protein corona formed in human serum (Table S1 and S2) and human plasma (Table S3 and S4). The properties of the CNMs and metal nanoparticles, along with the cited references, are listed in Tables S5 for human serum and S6 for human plasma. The program of meta-analysis was referred to reported procedures,[1] and performed using Artificial Intelligence tools. [Note: in comparison to other types of meta-analysis on protein corona composition across different families of nanoparticles, the number of references for CNMs is still rather limited as we focused only on human serum and human plasma, making the reliability of our results less systematic].

Discussion. In the human serum meta-analysis, CNMs and metal nanoparticles displayed distinct protein corona profiles. CNMs (e.g., graphene and NDs) showed a stronger binding of apolipoproteins (with ApoE being the most enriched protein), albumin, and coagulation factors, whereas metal nanoparticles absorbed more immunoglobulin and complement proteins. However, this tendency was not observed in the human plasma meta-analysis, as coagulation proteins and immunoglobulin dominate the protein corona of both CNMs and metal nanoparticles. This presumably due to the high content of coagulation factors in the plasma that mask the difference between the two types of nanomaterials.

Table S1. Top 10 prevalent proteins on CNM cohorts (human serum)

Protein	Class	CNM cohorts	Non-CNM cohorts	Diff[a]
Apolipoprotein E	Apolipoproteins	3	0	0.75
Transferrin	Other	2	0	0.50
Alpha-1-antitrypsin	Coagulation	2	0	0.50
Albumin	Other	2	1	0.30
Apolipoprotein A-IV	Apolipoproteins	2	1	0.30
Complement C9	Complement	1	0	0.25
Apolipoprotein C1	Apolipoproteins	1	0	0.25
Protein S	Coagulation	1	0	0.25

[a] Diff refers to the differences in proportion and/or frequency of specific corona proteins between CNM and Non-CNM cohorts.

Table S2. Top 10 prevalent proteins on Non-CNM cohorts (human serum)

Protein	Class	CNM cohorts	Non-CNM cohorts	Diff[a]
Apolipoprotein C-III	Apolipoproteins	0	3	-0.60
Ig lambda-2 chain C region	Immunoglobulins	0	3	-0.60
Ig lambda-7 chain C region	Immunoglobulins	0	3	-0.60
Complement C4-A	Complement	0	2	-0.40
Complement C4-B	Complement	0	2	-0.40
Ig mu chain C region	Immunoglobulins	0	2	-0.40
Apolipoprotein B-100	Apolipoproteins	0	2	-0.40
Ig kappa chain C region	Immunoglobulins	0	2	-0.40

[a] Diff refers to the differences in proportion and/or frequency of specific corona proteins between CNM and Non-CNM cohorts.

Table S3. Top 10 prevalent proteins on CNM cohorts (human plasma)

Protein	Class	CNM cohorts	Non-CNM cohorts	Diff[a]
Histidine-rich glycoprotein	Coagulation	4	1	0.52
Haptoglobin	Acute phase	3	0	0.50
Ig gamma-4 chain C region	Immunoglobulins	3	0	0.50
Ig lambda-2 chain C region	Immunoglobulins	3	0	0.50
Fibrinogen alpha chain	Coagulation	4	2	0.38
Vitronectin	Tissue leakage	3	1	0.36
Ig gamma-2 chain C region	Immunoglobulins	2	0	0.33
Ig gamma-1 chain C region	Immunoglobulins	4	3	0.24

[a] Diff refers to the differences in proportion and/or frequency of specific corona proteins between CNM and Non-CNM cohorts.

Table S4. Top 10 prevalent proteins on Non-CNM cohorts (human plasma)

Protein	Class	CNM cohorts	Non-CNM cohorts	Diff[a]
Actin beta	Other	0	4	-0.57
Integrin alpha-IIb	Coagulation	0	3	-0.43
Ig kappa chain C region	Immunoglobulins	0	3	-0.43
Talin-1	Other	0	3	-0.43
Integrin beta-3	Coagulation	0	3	-0.43
Myosin-9	Other	0	2	-0.29
Dermcidin	Other	0	2	-0.29
Filamin-A	Other	0	2	-0.29

[a] Diff refers to the differences in proportion and/or frequency of specific corona proteins between CNM and Non-CNM cohorts.

Table S5. References used for the meta-analysis in Table S1 and S2 (human serum)

Group	Nano cohort	References
CNM	MWCNTs	M. Sopotnik et al., Carbon 2015[2]
CNM	GO	
CNM	Graphene	V. Castagnola et al., Nat. Commun. 2018[3]
CNM	NDs	F. P. Martinez et al., Anal. Chem. 2019[4]
Au	AuNP	C. D. Walkey et al., J. Am. Chem. Soc. 2012[5]
Au	AuNP modified with tetramethylammonium	K. Saha et al., ACS Nano 2016[6]
Au	AuNP modified with *N,N,N*-trimethylbutan-1-aminium	
Au	AuNP modified with *N,N,N*-trimethylcyclohexanaminium	
Fe oxide	Iron oxide NP	Karina P. Garcla et al., ChemPlusChem 2017[7]

Table S6. References used for the meta-analysis in Table S3 and S4 (human plasma)

Group	Nano cohort	References
CNM	SWCNTs	Rebecca L. Pinals et al., Angew. Chem. Int. Ed. 2020[8]
CNM	GO	Massimiliano Papi et al, Nanoscale 2019[9]
CNM	GO	R. Di Santo et al, Front. Bioeng. Biotechnol. 2018[10]
CNM	NDs	Y. Zou et al., ACS Nano 2020[11]
CNM	ND coated with PEG	
CNM	ND coated with PG	
Au	AuNP coated with PEG	P. Chandran et al., Nanotoxicology 2017[12]
Au	AuNP coated with polyethyleneimine	
Au	AuNP coated with lipoic acid	
Fe oxide	Iron oxide NP coated with PEG	Alexandra G. Pershina et al., Colloids Surf., B. 2023 [13]
Fe oxide	Iron oxide NP coated with peptides	
Fe oxide	Iron oxide NP	Carmen Vogt et al., PLOS One 2015[14]
Fe oxide	Core shell iron oxide NP	

Table S7. The effects of CNM property on structural alteration of proteins and consequent biological outcomes

CNMs	Material properties	Structural alterations of proteins induced by CNMs	Biological consequences
MWCNTs	Tubular structure; High aspect ratio	BSA: distortion to secondary structures (α-helix, β-sheet, and β-turns)	Altered interaction with scavenger receptors on macrophage; Shifted uptake mechanism and efficiency
		AGP, IgG: unfolding	Activation of immune response
Graphene	Two-dimensional planar structure; Flat surface	BSA, IgE, myoglobin: transition of α-helix and β-sheet to β-coil/turn	Disturbance of protein native functions; Induced toxicity
		HP35: unfolding	—
		GB1: disruption of the α-helical structure	Compromised biosensing ability
DNDs	Ultrasmall size (< 5 nm); Graphitic layers; Charged species	Ins: increment of intermolecular β-sheet	—
		LYS: transition of α-helix to β-sheet and amorphous forms	Impaired enzymatic activity of LYS
		Intracellular proteins: transition of α-helical to β-sheet and β-turn-like structures	Oxidative stress to cells
CDs	Ultrasmall size (< 10 nm); High curvature	GOx: conformational change accompanied with reduction in α-helix content	Impaired thermal stability and enzymatic activity of GOx
CQDs		BLG: transition of isolated helices into coiled-coils and β-sheet	Disrupted binding of BLG to retinol ligand; Disable protein function

References

[1] A. Canchola, K. Li, K. Chen, A. Borboa-Pimentel, C. Chou, R. Dela Rama, C.-Y. Chen, X. Chen, M. Strobel, J. E. Riviere, N. A. Monteiro-Riviere, M. Wang, F. Zhang, Z. Lin, W.-C. Chou, *ACS Nano* **2025**, 19, 37633.

[2] M. Sopotnik, A. Leonardi, I. Križaj, P. Dušak, D. Makovec, T. Mesarič, N. P. Ulrih, I. Junkar, K. Sepčić, D. Drobne, *Carbon* **2015**, 95, 560.

[3] V. Castagnola, W. Zhao, L. Boselli, M. C. Lo Giudice, F. Meder, E. Polo, K. R. Paton, C. Backes, J. N. Coleman, K. A. Dawson, *Nat. Commun.* **2018**, 9, 1577.

[4] F. Perona Martinez, A. Nagl, S. Guluzade, R. Schirhagl, *Anal. Chem.* **2019**, 91, 9800.

[5] C. D. Walkey, J. B. Olsen, H. Guo, A. Emili, W. C. W. Chan, *J. Am. Chem. Soc.* **2012**, 134, 2139.

[6] K. Saha, M. Rahimi, M. Yazdani, S. T. Kim, D. F. Moyano, S. Hou, R. Das, R. Mout, F. Rezaee, M. Mahmoudi, V. M. Rotello, *ACS Nano* **2016**, 10, 4421.

[7] K. Pombo-García, C. L. Rühl, R. Lam, J. A. Barreto, C.-S. Ang, P. J. Scammells, P. Comba, L. Spiccia†, B. Graham, T. Joshi, H. Stephan, *ChemPlusChem* **2017**, 82, 638.

[8] R. L. Pinals, D. Yang, D. J. Rosenberg, T. Chaudhary, A. R. Crothers, A. T. Iavarone, M. Hammel, M. P. Landry, *Angew. Chem. Int. Ed.* **2020**, 59, 23668.

[9] M. Papi, V. Palmieri, L. Digiacomo, F. Giulimondi, S. Palchetti, G. Ciasca, G. Perini, D. Caputo, M. C. Cartillone, C. Cascone, R. Coppola, A. L. Capriotti, A. Laganà, D. Pozzi, G. Caracciolo, *Nanoscale* **2019**, 11, 15339.

[10] R. Di Santo, L. Digiacomo, E. Quagliarini, A. L. Capriotti, A. Laganà, R. Zenezini Chiozzi, D. Caputo, C. Cascone, R. Coppola, D. Pozzi, G. Caracciolo, *Front. Bioeng. Biotechnol.* **2020**, Volume 8 - 2020.

[11] Y. Zou, S. Ito, F. Yoshino, Y. Suzuki, L. Zhao, N. Komatsu, *ACS Nano* **2020**, 14, 7216.

[12] P. Chandran, J. E. Riviere, N. A. Monteiro-Riviere, *Nanotoxicology* **2017**, 11, 507.

[13] A. G. Pershina, A. M. Demin, N. A. Perekucha, O. Y. Brikunova, L. V. Efimova, K. V. Nevskaya, A. V. Vakhrushev, V. G. Zgoda, M. A. Uimin, A. S. Minin, D. Malkeyeva, E. Kiseleva, A. P. Zima, V. P. Krasnov, L. M. Ogorodova, *Colloids Surf., B.* **2023**, 221, 112981.

[14] C. Vogt, M. Pernemalm, P. Kohonen, S. Laurent, K. Hultenby, M. Vahter, J. Lehtiö, M. S. Toprak, B. Fadeel, *PLOS One* **2015**, 10, e0129008.
